# A Novel Biomarker Based on miRNA to Predict the Prognosis of Muscle-Invasive Bladder Urothelial Carcinoma

**DOI:** 10.1155/2019/2654296

**Published:** 2019-12-06

**Authors:** Yueyi Feng, Qingting Feng, Lingkai Xu, Yiqing Jiang, Fang Meng, Xiaochen Shu

**Affiliations:** ^1^Department of Epidemiology, School of Public Health, Medical College of Soochow University, Suzhou 215123, China; ^2^Department of General Surgery, Harrison International Peace Hospital, Hengshui 053000, China; ^3^Centre of Systems Medicine, Chinese Academy of Medical Sciences, Beijing 100730, China; ^4^Suzhou Institute of Systems Medicine, Suzhou 215123, China

## Abstract

Muscle-invasive bladder urothelial carcinoma (MIBC) is characteristic of high mortality and high recurrence. Distinguishing the prognostic risk of MIBC at the molecular level of miRNA expression is rarely performed and thus of great significance for the management and treatment of MIBC in clinics. Adaptive lasso Cox's proportional hazards model was used to explore the relationship between differential expression miRNAs (DEmiRNAs) and MIBC survival. Furthermore, we evaluated the epithelial-mesenchymal transition (EMT) score and immune infiltration abundance by exploring EMT signature genes and TIMER database, respectively. A total of 8 DEmiRNAs were detected to be associated with the survival rate of MIBC by using the lasso Cox algorithm. Through the linear combination of these 8 DEmiRNAs, we constructed a calculated marker, which could be used to distinguish the prognosis risk in both TCGA dataset (HR = 2.03, 95% CI = (1.47, 2.83)) and independent validation dataset (HR = 7.74, 95% CI = (1.05, 56.93)). Meanwhile, the constructed marker had reasonably high predictive values of the AUC (area under the curve) in the TCGA dataset and validation dataset being 0.73 and 0.63, respectively. In addition, we observed that the expression values of let-7c, miR-100, and miR-145 were associated with EMT score and the abundance of macrophage in tumor tissue as well. This newly identified risk score signature based on the combination of 8 miRNAs could significantly predict the prognostic risk of MIBC and might provide insight into immunotherapy and targeted therapy of MIBC.

## 1. Introduction

Bladder cancer is a commonly diagnosed malignant tumor arising from the tissues of the urinary system, with around 550,000 new cases and 200,000 deaths being reported worldwide in 2018 [1]. About 1 out of 4 bladder cancer cases were diagnosed as muscle-invasive bladder cancer (MIBC) when cancer cells have gone through the bladder lining and are present in the detrusor muscle [2, 3]. MIBC has strong invasiveness and is more prone to distal metastasis and recurrence, which brings about a poor 5-year survival of around 50% [4]. At present, radical cystectomy of the bladder in conjunction with neoadjuvant therapy is still the first-line treatment for MIBC [5]. However, most MIBC cases are less sensitive to neoadjuvant chemotherapy based on cisplatin [4, 6]. The 5-year survival rates of MIBC have not improved as much as other cancers during decades [7]. Identifying MIBC prognostic biomarkers at the molecular level will thus be of great value in distinguishing MIBC cases with different risks, developing individualized clinical therapies, and improving survival rate.

MicroRNAs (miRNAs) comprise a class of small, endogenous noncoding RNA with short nucleotide length (20–25 nt) that are involved in the process of cell proliferation, differentiation, migration, and apoptosis by regulating the expression and translation of target mRNA [8, 9]. It has been observed that the abnormal expression of miRNA can destroy the RNA network in cancer cells and promote the occurrence and development of tumors, especially urinary tumors [10–12]. In addition, some studies have shown that miRNAs also regulate the epithelial-mesenchymal transition (EMT) process, which enhances the invasiveness of cancer and enriches the abundance of stem cells in tumor tissues [13]. Although miRNA has been widely reported as a tumor marker, so far, there have been few reports about miRNA and the survival of MIBC. The available reports are based on small number of patients or single miRNA [14]. To our knowledge, there is no study integrated miRNA, EMT, and immune infiltration status, with comprehensive prognostic factors of MIBC so far.

In this study, we analyzed the level 3 miRNA data from the Cancer Genome Atlas (TCGA) database (https://portal.gdc.cancer.gov/) to screen differential expression miRNA (DEmiRNA) between tumor and normal tissues. Among DEmiRNAs, we used the lasso Cox algorithm to identify a group of miRNAs which were associated with overall survival (OS), constructed a risk model based on 8 miRNAs, and verified it in an independent external dataset from Gene Expression Omnibus (GEO) database. Furthermore, we used time-dependent receiver operating characteristic (ROC) curve to evaluate the predictive value of the five-year survival rate of the model in two datasets, respectively. Also, we explored the potential association of miRNA with EMT and with the abundance of immune cell infiltration in tumor tissues.

## 2. Materials and Methods

### 2.1. Data Collection and Preprocessing

The expression profiles of miRNA (Illumina HiSeq) and the corresponding clinical information (Genomic Data Commons) of patients diagnosed with muscle-invasive bladder urothelial carcinoma (MIBC) were downloaded from TCGA and GEO. Patients in the TCGA dataset were excluded from the present study if they met any of the following criteria: (1) the pathological grade was lower than pT2; (2) the first diagnosis cancer was not bladder cancer; (3) received treatment before being involved in the TCGA cohort; (4) NMIBC (non-muscle-invasive bladder cancer) patients progressed to MIBC after the tissue was obtained; and (5) the follow-up status and times were not available. A total of 12 patients who had previously been diagnosed with other cancers or had previously received treatment or not meet study protocol were excluded from the TCGA datasets by reading annotation files. Totally, 392 tumor samples and 17 normal samples from the TCGA datasets were included in the follow-up analysis [15]. The GSE84525 dataset from GEO was utilized to validate the findings from TCGA, which included 62 MIBC samples [16]. In addition, we also downloaded mRNA data to evaluate the status of EMT (epithelial-mesenchymal transition) and immune cell infiltration from the TCGA database.

### 2.2. Screening Differentially Expressed miRNA

The “DESeq2” R package was applied to screen the DEmiRNAs between normal and MIBC samples from the TCGA dataset [17]. For low-count miRNAs, we used the default filter parameters (minmu: lower bound on the estimated count for fitting gene-wise dispersion) of the “DESeq” function in the DESeq2 package to filter them (the default value was 0.5). DEmiRNAs were selected according to the following criterion: the *P* (FDR) value less than 0.01 and the absolute value of log_2_(fold change) (logFC) more than or equal 2. In the TCGA dataset, the expression of miRNAs was normalized by carrying out the variance stabilizing transformation (VST), and the “blind” parameter was set to FALSE. Finally, in TCGA and GEO datasets, the expression value of miRNAs was transformed by log_2_(*x* + 1).

### 2.3. Model Development and Validation

First, we used univariate Cox regression to screen DEmiRNA in the TCGA dataset following the criterion that *P* value was less than 0.05 and the follow-up times were limited to 60 months. We screened 26 miRNAs which were relevant with MIBC prognosis from 129 DEmiRNAs for further analysis. Second, adaptive lasso penalty Cox regression (ALasso) was used to further screen the more important and stable DEmiRNA where the *λ* value was obtained by a 10-fold cross-validation [18]. Third, we obtained the coefficient (*β*) of every DEmiRNAs, and the prognostic risk score model for predicting overall survival was constructed according to the following formula:

(1) $$\begin{array}{c} \text{Risk Score}=\beta_{1}*\;\exp_{1}+\beta_{2}*\;\exp_{2}+\cdots +\beta_{n}*\;\exp_{n}, \end{array}$$

where *β*: the ALasso Cox regression coefficient and exp: log_2_(the expression of miRNA + 1). Fourth, the patients from TCGA were categorized into high- and low-risk groups using the optimal cutoff value of the risk score, which was performed by using the X‐tile software (version 3.6.1). The survival differences between the two groups were compared by log-rank test. However, before the comparison process, the groups were tested for the proportional hazard assumption, and if the assumption was not satisfied, it was presented in two strata. Meanwhile, we validated the risk score model in the GEO dataset using the same cutoff value. Fifth, the time-dependent receiver operating characteristic (ROC) curve was used to evaluate the predictive value of the survival rate of the constructed model [19]. In addition, we also evaluated the differences of progress-free interval (PFI) between two groups in TCGA datasets.

### 2.4. Evaluation of EMT Score and Immune Cell Infiltration

We calculated the EMT score to evaluate the EMT status of each patient using previously reported EMT (epithelial-mesenchymal transition) signature genes, that is, the average expression of mesenchymal signature genes minus the average expression of epithelial signature genes [15, 20]. We also evaluated the infiltration abundance of B cells, CD4 T cells, CD8 T cells, neutrophil, macrophage, and dendritic in tumor tissue from RNA-seq expression data by TIMER network tool which was a deconvolution method [21].

### 2.5. Consensus Clustering

All samples from TCGA were clustered based on miRNA expression value of model by consensus clustering using ConsensusClusterPlus R package and the distance metric using Pearson distance [22].

### 2.6. Gene Function Analysis

In order to explore the potential functions of miRNA in the risk model, we retrieved targeted mRNAs which were regulated by miRNA of risk model from miRTarBase, miRDB, and TargetScan databases by miRBase ID [23–25]. We used “clusterProfiler” R package to perform functional enrichment analysis for these targeted miRNAs which included KEGG (Kyoto Encyclopedia of Genes and Genomes) pathway and GO (Gene Ontology) biological process [26]. The criterion was adjusted *P* value less than 0.05.

## 3. Results

### 3.1. Patient Characteristics

In TCGA datasets, a total of 392 patients were included in this study after preprocessing. The demographic and clinical characteristics are listed in Table 1, while in GEO datasets, a total of 62 patients were included in the present study in which only information on age (median age: 68) and survival was available.

### 3.2. Screening Differentially Expressed miRNA

**Figure 1:**
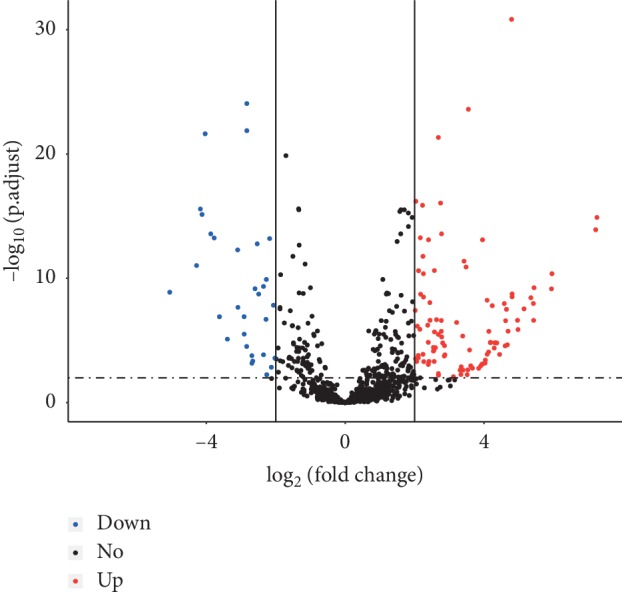
Volcano plot of DEmiRNAs. The *x*-axis indicates log_2_(fold change), and *y*-axis indicates–log_10_(adjust *P* value). Blue, red, and black represent upregulation miRNAs, downregulation miRNAs, and nondifferential expression miRNAs, respectively.

According to the criteria described in the Method section, a total of 129 DEmiRNAs were identified in this study which included 98 upregulated and 31 downregulated ones (Figure 1).

### 3.3. Construction of Risk Score Model

First, we used univariate Cox regression to explore the potential association of DEmiRNAs with overall survival in the TCGA dataset, which generated a total of 26 miRNAs with *P* < 0.05. Next, adaptive lasso Cox regression was employed to further select eight stable miRNA combinations whereby we constructed a risk model according to the formula as follows:

(2) $$\begin{array}{c} \text{Risk Score}=\;0.081*\;\exp_{\text{let}-7c}+0.056*\;\exp_{miR-100}+0.155*\;\exp_{miR-145}+0.064*\;\exp_{miR-519c}+0.088*\;\exp_{miR-615}+0.224*\;\exp_{miR-33b}+0.092*\;\exp_{miR-1251}-0.039*\;\exp_{miR-138}. \end{array}$$

**Figure 2:**
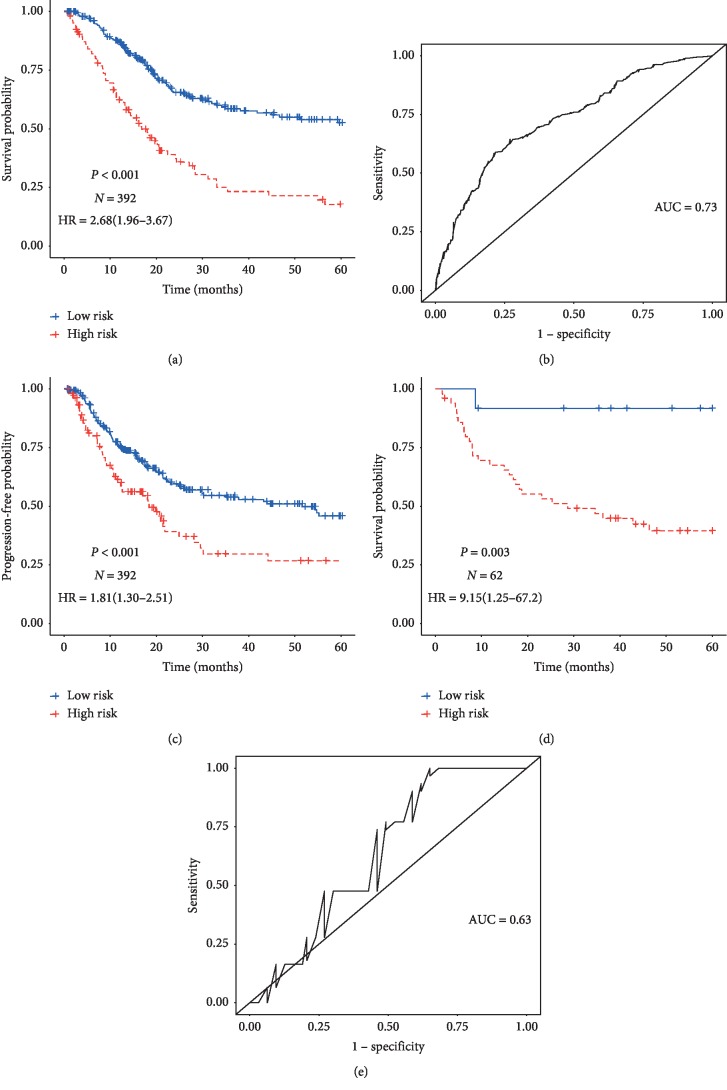
Kaplan–Meier curves and time-dependent receiver operating characteristic curves of miRNA signature. In the TCGA dataset, (a, b) the endpoint is overall survival and (c) the endpoint is progress-free interval. In the validation dataset, (d) the endpoint is overall survival; (e) time-dependent receiver operating characteristic curves of miRNA signature.

Furthermore, we calculated the risk score for each patient and used 4.91 as the optimal cutoff value to divide the patients into high-risk and low-risk groups which was performed by using X-tile software. A worse prognosis was observed for the high-risk group (HR = 2.03, 95% CI = (1.47, 2.83)) compared to the low-risk group after multiple adjustments (Table 2). The Kaplan–Meier curve also showed a better prognosis for lower risk group (Figure 2(a)). Additionally, the time-dependent ROC curve shows that the risk model had good forecasting ability, i.e., a AUC (area under the curve) of 0.73 (Figure 2(b)). We explored the difference in progress-free interval (PFI) between the two groups of patients which showed a shorter PFI in the high-risk group (Figure 2(c)). Additionally, we compared clinical characteristics between different groups by risk model using logistics regression. More advanced pathological stage of cancer was observed among patients in the low-risk group by the risk model (OR = 0.54, 95% CI = (0.40–0.73)).

### 3.4. Risk Model Validation

An external independent dataset was adopted to validate the risk model. In this external dataset, patients were divided into two groups using the same cutoff value which was defined in the TCGA dataset. Consistent with the TCGA dataset, the high-risk group (HR = 7.74, 95% CI = (1.05, 56.93)) had unfavorable prognosis than the low-risk group (Table 2, Figure 2(d)). The predictive performance of the risk model was also evaluated (AUC = 0.63, Figure 2(e)).

### 3.5. Epithelial-Mesenchymal Transition

**Figure 3:**
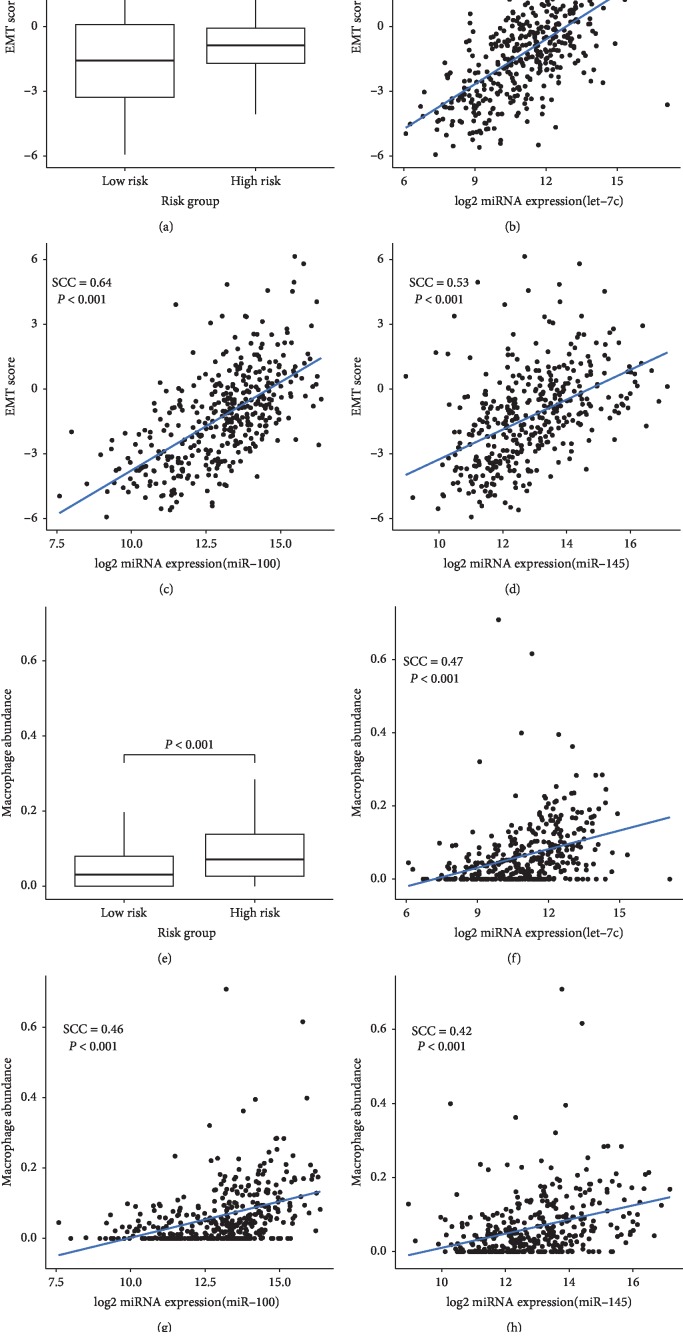
(a) The boxplot of EMT score for different risk groups. Plot of Spearman's correlation between EMT score and the expression of (b) let-7c, (c) miR-100, and (d) miR-145. (e) The boxplot of the abundance of macrophage in tumor tissue for different risk groups. Plot of Spearman's correlation between the abundance of macrophage and the expression of (f) let-7c, (g) miR-100, and (h) miR-145.

Considering the important role of epithelial-mesenchymal transition (EMT) progress played in the occurrence and development of MIBC, we explored the underlying relationship between the expression level of miRNA and EMT scores. We observed that patients in the high-risk group had higher EMT scores than those in the low-risk group (*P*=0.005, Figure 3(a)). We also evaluated the correlation between miRNAs and EMT. We found that three miRNAs were positively correlated with EMT, that is, let-7c (SCC (Spearman's correlation coefficient) = 0.61, Figure 3(b)), miR-100 (SCC = 0.64, Figure 3(c)), and miR-145 (SCC = 0.53, Figure 3(d)).

### 3.6. Immune Cell Infiltration

**Figure 4:**
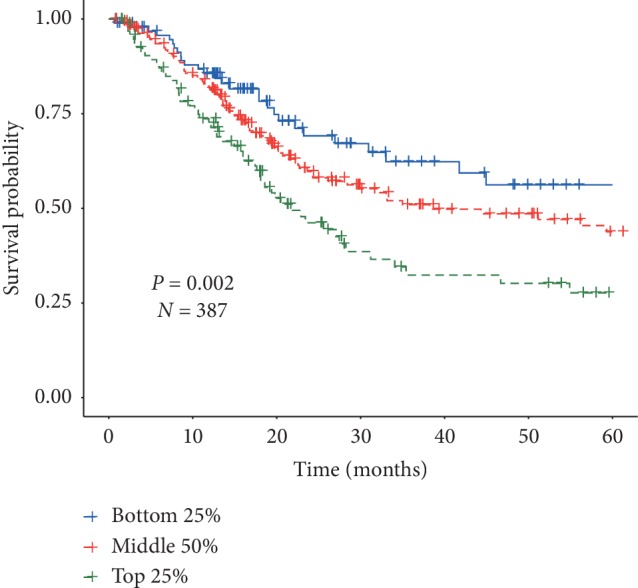
Kaplan–Meier estimates of probability of overall survival among different infiltration abundance of macrophage.

Studies have shown that infiltrating immune cells are participating in the inflammatory response of tumor microenvironment, which is of great significance to the prognosis of cancer [27]. We used RNA-seq data to evaluate the infiltration abundance of 6 immune cells in 387 patients and found that patients in the high-risk group had higher macrophage infiltration abundance (*P* < 0.001, Figure 3(e)). Next, the potential correlation was explored by Spearman's rank correlation between immune cell infiltration abundance (ICIB) and several miRNAs, that is, let-7c (SCC = 0.47, Figure 3(f)), miR-100 (SCC = 0.46, Figure 3(g)), and miR-145 (SCC = 0.42, Figure 3(h)). Additionally, we compared the overall survival rates between different infiltration abundance (top 25%, middle 50%, and low 25%). Worse prognoses were observed among higher macrophage infiltration abundance (Figure 4).

### 3.7. Subtype Identification

**Figure 5:**
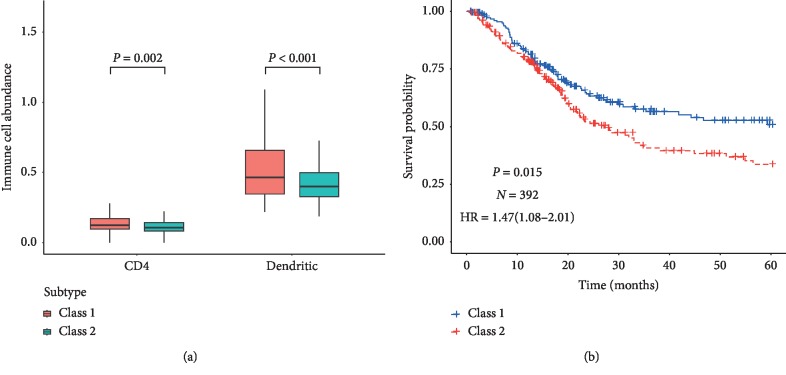
(a) The boxplot of the abundance of CD4 T cell and dendritic in tumor tissue for different MIBC subtypes. (b) Kaplan–Meier curves of different MIBC subtypes; the endpoint is overall survival.

392 samples from TCGA datasets were divided into two categories by consensus clustering of 8 miRNA expression values. By comparing the ICIB between two classes, we noticed that the infiltration level of CD4 T cells and dendritic cells in class 1 was higher (Table 3, Figure 5(a)). And the class 2 (HR = 1.47, 95% CI = (1.08, 2.01)) had unfavorable prognosis than class 1 (Figure 5(b)). Therefore, we defined two MIBC immune subtypes according to the infiltration of immune cell. We, additionally, observed that the common patients in the same prognosis groups were 70 (class 2, high-risk groups, poor prognosis) and 157 (class 1, low-risk groups, better prognosis) in consensus clustering and risk model group.

### 3.8. Gene Function Analysis

We performed the function analysis for target genes from three different databases (Supplementary file, [Supplementary-material supplementary-material-1]). According to the criterion (adjusted *P* value < 0.05), GO (Gene Ontology) enrichment analysis showed that targeted genes enriched 622 (miRDB), 1296 (miRTarBase), and 801 (TargetScan) GO terms for the three databases above, respectively (Supplementary file, Tables [Supplementary-material supplementary-material-1], [Supplementary-material supplementary-material-1], [Supplementary-material supplementary-material-1]). We found 230 common terms in these three databases, indicating that miRNA may be involved in regulation of cell morphogenesis, epithelial to mesenchymal transition, stem cell differentiation, and regulation of macroautophagy (Supplementary file, [Supplementary-material supplementary-material-1]). Pathway analysis showed that three databases enriched 72 (miRDB), 77 (miRTarBase), and 30 (TargetScan) pathways, respectively (Supplementary file, [Supplementary-material supplementary-material-1], [Supplementary-material supplementary-material-1], [Supplementary-material supplementary-material-1]). Among the 10 same pathways included MAPK signaling pathway, signaling pathways regulating pluripotency of stem cells, and erbB signaling pathway (Supplementary file, [Supplementary-material supplementary-material-1]).

## 4. Discussion

Muscle-invasive bladder cancer (MIBC) is highly heterogeneous and its prognosis has not improved in the past decades [7]. Molecular and genetic advances could provide perspectives on potential therapeutic targets for MIBC based on novel biomarkers, from which the prognosis of MIBC will be benefited. In recent years, more miRNA data are available for various cancers. miRNA profiling is becoming an important tool for cancer prognosis since the pattern of miRNA expression can be correlated with clinical characteristics of cancer under the possible mechanisms of proliferation, apoptosis, invasion/metastasis, and angiogenesis. Increasing evidence showed that the abnormal expression of miRNA plays an important role in the metastasis and development of cancer [27, 28].

In the present study, a total of 8 miRNA associated with overall survival were screened from 129 differentially expressed miRNAs by using univariate Cox regression and adaptive lasso method. We constructed a risk score model by linearly combining 8 miRNAs and validated this model in an independent external dataset. Furthermore, we found that three miRNAs in risk model were associated with immune cell infiltration abundance (ICIB) and epithelial-mesenchymal transition score. We also observed significant differences in ICIB and EMT score according to different risk groups. In addition, gene function analysis showed that the DEmiRNAs included by the risk model were mainly involved in biological processes or pathways such as stem cell differentiation, cell growth, and axon guidance.

Macrophages, a driving factor of inflammatory reaction, were the major players of tumor microenvironment [29]. Studies have shown that the number of macrophages was associated with the poor prognosis of cancer, which was supported by our findings [30]. In our study, we observed that the estimated MIBC risk for patients (high vs. low) by the risk model was significantly associated with macrophage abundance and prognosis as well. The potential biological mechanism may be related to that tumor cells stimulate macrophages to secrete a large number of inflammatory factor such as interleukin-10 (IL-10) and tumor necrosis factor-*α* (TNF-*α*) and further to promote the expression of programmed cell death ligand 1 (PD-L1) and eventually make cancer cells escape from the attack of T cells [31]. Other mechanisms could be that macrophages regulated the effects of Tregs (regulatory T cells) by promoting the synthesis of prostaglandin E2 (PGE2) to promote immunosuppressive response or the participation of miRNA in the immune escape process [32, 33].

Epithelial-mesenchymal transition (EMT) is a process in which differentiated epithelial cells lose their epithelial characteristics and change into a motile, mesenchymal phenotype, which makes cells to gain strong ability to invade, proliferate, and escape apoptosis and to participate in immunosuppressive response [34]. We noted that the high expression of miR-100, miR-145, and let-7c in MIBC promoted the process of EMT. A study of breast cancer showed that miR-100 could induce the EMT process by regulating the expression of CDH1 (cadherin 1) through SMARCA5 [35]. Other studies found that miR-145 regulated TGF-*β*1- (transforming growth factor-*β*1-) mediated EMT progress to enhance the invasiveness of cancer cells by targeting SMAD5 [36, 37]. In contrast to our results, let-7c has been reported to be a tumor inhibitor and inhibited the process of EMT for other cancers. The inconsistent results could be either due to an endogenous competition relationship between the target mRNA of let-7c and some long noncoding RNA or caused by the tissue-specific expression of miRNA [38, 39].

Previous studies have shown that miR-615 can increase the level of Mcl-1 protein and promote the proliferation and metastasis of cancer cells and the ability of antiapoptosis by inhibiting the expression of CELF2 in cancer cells [40, 41]. In addition, miR-615 and miR-1251 jointly targeted IGF1R (insulin-like growth factor 1 receptor) and participated in the reproductive process of cancer cells through phosphoinositide 3-kinase-Akt pathway and the shc-ras-MAPK pathway [42, 43].

Several other markers were observed to be relevant to tumorigenesis in different mechanisms. The high expression of miR-33b suppresses the transcription and translation of CDKN1A (cyclin-dependent kinase inhibitor 1A), which makes cancer cells to acquire stem cell characteristics, enhance the resistance of cancer cells to cisplatin drugs, and participate in the regulation of Tregs cells in tumor immune microenvironment [44–46]. In bladder cancer, miR-138 binds to the 3′TUR sequence of ZEB2 (zinc finger E-box binding homeobox 2), regulates the expression and phosphorylation of vimentin and e-cadherin, reverses the EMT process, and enables cancer cells to obtain epithelial characteristics [47]. In addition, it has been reported that miR-138 improved the sensitivity of tumor patients to gefitinib by silencing the G protein-coupled receptor (GPCR) [48]. To date, the abnormal expression of miR-519c has not been reported to be related to MIBC prognosis. However, a recent study reported that miR-519c could worsen the prognosis of hepatocellular carcinoma (HCC) by negatively regulating the expression of BTG antiproliferation factor 3 (BTG3) which could promote growth and metastasis of HCC [49]. In addition, miR-519c was found to be involved in the expression and regulation of genes related to chemotherapeutic sensitivity of tumor cells, such as ATP binding cassette subfamily G member 2 (ABCG2) [50].

In this study, we constructed a novel predictive biomarker for MIBC prognosis based on the expression of multiple miRNAs based on a series of strict criteria. Compared to available relevant studies using TCGA and GEO data to date, the strengths of our study mainly focused on the following perspectives. First, our study subjects were strictly restricted to MIBC which has a higher risk of clinical death than NMIBC which was included as subjects in most other studies. Meanwhile, we excluded patients in TCGA cohort from the present study by carefully reading attachment annotation, which were considered not suitable for MIBC study by TCGA working groups in order to achieve a better representation. Second, we, for the first time, investigated the relationship between the expression of miRNAs and EMT progress to see the possible role of EMT process as a potential mechanism in the MIBC prognosis and consequently found the differential EMT status in different risk groups. We also explored the relationship between signature miRNAs and EMT (let-7c, miR-100, and miR-145). Third, we identified the MIBC immune subtypes by miRNAs and evaluated the effect of immune cell abundance on the prognosis of MIBC to explore the impact of immune microenvironment on MIBC prognosis, which had not been reported to our knowledge, too. Fourth, adaptive lasso algorithm model was used to reduce the false-positive rate compared with the classical Cox screening method. However, better ROC values could be achieved when more miRNA and related data became available besides the methodological improvements. Other limitations existed. First, our data were abstracted from the public database, where treatment information was not available. Furthermore, the external independent dataset for verification lacks clinical information such as pathological grade. In addition, the EMT status in 4 patients and the ICIB in 5 patients could not be evaluated.

## 5. Conclusions

In summary, we analyzed miRNA data and clinical data from 392 patients diagnosed with MIBC from the TCGA project. A combination of 8 miRNAs which were constructed and validated independently to be related to MIBC survival could be suggestive of potential prognostic markers in clinics and might provide perspectives for therapeutic target. We also found the association of three miRNAs with immune cell infiltration abundance and EMT process, which may shed light on the underlying mechanism for MIBC prognosis and recurrence.

## Figures and Tables

**Table 1:** Characteristics of the TCGA dataset.

Characteristics	Number	%
All	392	100
Age		
Mean	68 ± 10.7	—
Gender		
Female	104	26.5
Male	288	73.5
Race		
White	312	79.6
Asian Black or African	42	10.7
American	22	5.6
Unknown	16	4.1
Stage		
Stage II	127	32.4
Stage III	137	34.9
Stage IV	128	32.7
Grade		
High	370	94.4
Low	20	5.1
Unknown	2	0.5
Survival		
Dead	163	41.6
Alive	229	58.4

**Table 2:** Survival analysis for risk model with OS as endpoints in the TCGA dataset and validation dataset.

	TCGA dataset (*N* = 392)						Validation dataset (*N* = 62)					
	Univariate			Adjusted^a^			Univariate			Adjusted^b^		
Variable	HR	95% CI	*P*	HR	95% CI	*P*	HR	95% CI	*P*	HR	95% CI	*P*
Age	1.03	1.02–1.05	<0.001	1.02	1.01–1.04	0.009	1.07	1.02–1.11	0.002	1.06	1.02–1.11	0.005
Gender^c^	0.88	0.63–1.24	0.463	0.84	0.6–1.18	0.311	—	—	—	—	—	—
Stage^d^	1.8	1.47–2.21	<0.001	1.61	1.31–1.98	<0.001	—	—	—	—	—	—
Risk^e†^	2.68	1.96–3.67	<0.001	2.03	1.47–2.83	<0.001	9.15	1.25–67.2	0.03	7.74	1.05–56.93	0.045

^a^Adjusted for age, pathological stage, and gender. ^b^Adjusted for age. ^c^Female (ref) vs. male. ^d^Stage II (ref) vs. stage III vs. stage IV. ^e^High risk vs. low risk (ref). ^†^Proportional risk assumptions were met.

**Table 3:** Immune cell infiltration abundance of two subtypes.

Immune cell type	Class 1		Class 2		*P* ^b^
	Median	IQR^a^	Median	IQR^a^	
B cell	0.073	0.045–0.098	0.074	0.048–0.103	0.463
CD4 T cell	0.124	0.097–0.171	0.106	0.084–0.142	0.002
CD8 T cell	0.156	0.108–0.217	0.151	0.104–0.207	0.589
Neutrophil	0.112	0.073–0.168	0.093	0.068–0.133	0.079
Macrophage	0.035	0–0.087	0.045	0.003–0.097	0.257
Dendritic	0.465	0.346–0.657	0.400	0.329–0.498	0.001

^a^IQR : interquartile range (Q_25_-Q_75_). ^b^*P* value is obtained by Shapiro–Wilk test.

## Data Availability

The data used to support the findings of this study are available from the corresponding author upon request.

## Supplementary Materials

Supplementary Materials Table S1: targeted mRNA of DEmiRNAs—targeted mRNAs of DEmiRNAs from miRDB, miRTarBase, and TargetScan. Table S2: miRDB GO function—Gene Ontology enriched analysis of target mRNA of DEmiRNA. The target mRNA obtained from miRDB database. Table S3: miRTarBase GO function—Gene Ontology enriched analysis of target mRNA of DEmiRNA. The target mRNA obtained from miRTarBase database. Table S4: TargetScan GO function—Gene Ontology enriched analysis of target mRNA of DEmiRNA. The target mRNA obtained from TargetScan database. Table S5: common GO terms—intersection of GO analysis results of three databases. Table S6: miRDB KEGG pathways—KEGG pathway enriched analysis of target mRNA of DEmiRNA. The target mRNA obtained from miRDB database. Table S7: miRTarBase KEGG pathways—KEGG pathway enriched analysis of target mRNA of DEmiRNA. The target mRNA obtained from miRTarBase database. Table S8: TargetScan KEGG pathways—KEGG pathway enriched analysis of target mRNA of DEmiRNA. The target mRNA obtained from TargetScan database. Table S9: common KEGG pathways—intersection of KEGG pathway analysis results of three databases.
